# The Neural Basis of Maternal Bonding

**DOI:** 10.1371/journal.pone.0088436

**Published:** 2014-03-04

**Authors:** Ming Wai Wan, Darragh Downey, Hilary Strachan, Rebecca Elliott, Steve R. Williams, Kathryn M. Abel

**Affiliations:** 1 Centre for Women's Mental Health, Institute of Brain, Behaviour and Mental Health, University of Manchester, Manchester, United Kingdom; 2 Neuroscience and Psychiatry Unit, Institute of Brain, Behaviour and Mental Health, University of Manchester, Manchester, United Kingdom; 3 Bolton National Health Service Foundation Trust, Bolton, Lancashire, United Kingdom; 4 Centre for Imaging Science, University of Manchester, Manchester, United Kingdom; University of Bologna, Italy

## Abstract

**Background:**

Accumulating evidence suggests that mothers show a different pattern of brain responses when viewing their own compared to other infants. However, there is inconsistency across functional imaging studies regarding the key areas involved, and none have examined relationships between brain and behavioural responses to infants. We examined the brain regions activated when mothers viewed videos of their own infant contrasted with an unknown infant, and whether these are associated with behavioural and self-reported measures of mother-infant relations.

**Method:**

Twenty right-handed mothers viewed alternating 30-sec blocks of video of own 4–9 month infant and an unfamiliar matched infant, interspersed with neutral video. Whole brain functional magnetic resonance images (fMRI) were acquired on a 1.5T Philips Intera scanner using a TR of 2.55 s. Videotaped mother-infant interactions were systematically evaluated blind to family information to generate behavioural measures for correlational analysis.

**Results:**

Enhanced blood oxygenation functional imaging responses were found in the own versus unknown infant contrast in the bilateral precuneus, right superior temporal gyrus, right medial and left middle frontal gyri and left amygdala. Positive mother-infant interaction (less directive parent behaviour; more positive/attentive infant behaviour) was significantly associated with greater activation in several regions on viewing own versus unknown infant, particularly the middle frontal gyrus. Mothers' perceived warmth of her infant was correlated with activations in the same contrast, particularly in sensory and visual areas.

**Conclusion:**

This study partially replicates previous reports of the brain regions activated in mothers in response to the visual presentation of their own infant. It is the first to report associations between mothers' unique neural responses to viewing their own infant with the quality of her concurrent behaviour when interacting with her infant and with her perceptions of infant warmth. These findings provide support for developing fMRI as a potential biomarker of parenting risk and change.

## Introduction

A mother's emotional tie to her infant is especially important for ensuring infant survival and healthy psychosocial development [1]–[2]. Human infants are born with facial features and other physical and behavioural characteristics which elicit adult proximity and care [3]. Moreover, while an infant is preverbal, the mother may rely more on viewing facial emotions to understand her child's emotions than at any other time. Exposing a mother to visual stimuli of her child may evoke a pattern of brain response that is different to or stronger than when viewing other children. This difference in brain response when viewing her own infant (compared with an unknown infant) may be characteristic of the special mother-infant emotional bond which, on the one hand, may underlie responsive parenting, and – on the other hand – may be lacking in mothers who struggle with feeling emotional closeness with their infant, and subsequently, with parenting.

Combined with sophisticated behavioural paradigms, functional magnetic resonance imaging (fMRI) has enabled us to begin to understand the neurobiological basis of the mother-infant bond *in vivo*. Studies have shown greater activation in specific brain regions when healthy mothers view images of their own infant compared with an unknown infant [4]–[9]. A few studies have used video stimuli [10]–[12]; these are likely to be more ecologically valid (or naturalistic) than static visual images. Swain et al. attempted to integrate functional imaging studies of maternal responsiveness with studies in animals to develop a ‘parental brain model’, characterised as a hypothalamic-midbrain-limbic-paralimbic-cortical circuit [13], [14].

However, across the relevant nine studies to date, there is low consensus with respect to the brain regions recruited in mothers in response to the visual stimulus of their own infant. The regions most often implicated (prioritising the most methodologically similar research to the current study) are the right middle occipital gyrus [7], [12], right superior frontal gyrus [5], [7], [10], amygdala (left [4], [7], [10], [12] and right [4], [10]), left orbitofrontal cortex [11], [12], and thalamus [5], [9], [11], based on our review of the coordinates of previous studies. Several reasons may explain this lack of consistency. Firstly, maternal emotional bonding is highly complex comprising primarily affective, but also cognitive and behavioural processes [15], which are also likely to be influenced by infant, mother and dyadic (mother-infant) characteristics [16]–[18]. Secondly, the mother's brain processing unique to viewing her own infant is likely to be sensitive to subtle methodological differences between studies. Thirdly, few studies have been directly replicated, so they differ in fMRI paradigm, infant stimuli and instructions given to the parent, while the lag in the fMRI measure of blood flow change means that highly transient or more sustained emotion may be missed. Fourthly, sample characteristics such as infant age vary across studies, and sample sizes have typically been small. Finally, region-of-interest (ROI) analyses have been increasingly used and, while offering better sensitivity over whole-brain analyses, they necessarily test specific research questions, which precludes comparison between studies.

To date, one study has attempted to link observed maternal behaviour with fMRI activation to own infant visuals [10]. Mothers were classified as either synchronous (N = 13) or intrusive (N = 10) to test the hypothesis of the differential recruitment of the nucleus accumbens and the amygdala in response to viewing videos of own versus an unknown infant, which they found support for. This forms the first evidence supporting the notion that sensitive (or synchronous) mothers show a different neural pathway in response to viewing own infant than less sensitive (and specifically, intrusive) mothers. However, mothers did not view their infant versus an unknown infant *per se*, Rather, they viewed 2-mins solitary infant play and 2-mins of themselves interacting with their infant, of which the latter was used as a basis for group assignment (synchronous or intrusive), as well as for the correlational measure. The ‘unknown infant’ comparison stimuli (viewed in typical and ‘pathological’ interactions) were consistent across participants, irrespective of the quality of the index mother's own interaction. Thus, the own infant and other infant stimuli differed in many ways beyond familiarity; subsequently, the degree of difference varied between participants. Since evidence suggests that it is the *degree* of maternal sensitivity which plays an important role in the child's long-term psychological development [19]–[21], directly linking the extent of neural responses to sensitivity of maternal behavioural response may provide more developmentally meaningful data than constraining maternal behaviour within two classifications [10].

This study set out to replicate our original smaller study of mothers [12] in a larger sample, carefully matching own and unknown infant video stimuli. The aim was to explore: (1) which maternal brain regions were activated in response to viewing own versus a matched unknown infant; and (2) whether greater magnitude of activation in any particular regions is associated with quality of mother-infant relations. Given the low consensus on the key areas activated in response to viewing own infant versus an unknown infant across the nine relevant studies, we generally favoured a whole brain analytic approach over making regionally-specific *a priori* hypotheses. However, amygdala response is one of the most consistent findings [4], [7], [10], [12], including in our previous study [12], and we therefore performed an ROI analysis for this region.

## Methods

### Participants

Twenty healthy, right-handed mothers of 4- to 10-month-old healthy infants were recruited from the Manchester region in the UK through free local advertising in order to obtain a sample from a range of socioeconomic backgrounds (*Table 1*). All participants reported normal vision or vision corrected to normal. No infants had been separated from the mother since birth, and 60% were firstborns (*Table 1*). Most (75%) mothers had breast fed for some or all of the time since the birth, and 9 (45%) mothers were currently breastfeeding. All but one mother lived with their partner. One mother was taking medication (an antidepressant, though not during pregnancy) and had scored >11 (i.e. 14) on the Edinburgh Postnatal Depression Scale [22], a 10-item self-report measure designed to screen for postnatal depression. The study was approved by the National Health Service North West Multi-Site Research Ethics Committee (ref: 05/MRE08/69); participants gave signed informed consent and were compensated for participation.

**Table 1:** Sample characteristics.

	Mean [SD]	Range
Maternal age (years)	32 [6.84]	20–43
Infant age (months)	6.20 [1.64]	4–10
EPDS score	5.85 [3.82]	0–14
	Frequency	Frequency
Ethnicity	Caucasian	14 (70%)
	Asian/black/mixed	6 (30%)
Highest qualification	High school (GCSE)	7 (35%)
	Post-secondary vocational	5 (25%)
	Post-secondary academic	2 (10%)
	Degree/diploma	6 (30%)
Housing status	Owner occupier	15 (75%)
	Tenant: Housing association or council	4 (20%)
	Resident with employer	1 (5%)
Living with partner	Yes	19 (95%)
Occupation	Professional/managerial	5 (25%)
	Skilled non-manual or manual	6 (30%)
	Semi-skilled/unskilled	3 (15%)
	Homemaker/student	6 (30%)
Parity	1	12 (60%)
	2	4 (20%)
	3+	4 (20%)
Infant gender	Boy	7 (35%)
Breastfeeding history	Yes/sometimes	15 (75%)
	No	5 (25%)
Current breastfeeding	Yes	9 (45%)

### Procedure

The initial session, approximately 3 weeks before the scanning session, involved capturing two video recordings: 2-mins of their infant's head and shoulders (for later editing for the fMRI activation paradigm), and 6-mins mother-infant play interaction in the mother's home or on university premises (as the mother preferred), followed by questionnaire measures and participant background information. The interval between sessions allowed for video editing to create the fMRI stimuli. In the scanning session, the infant was left with another carer or was looked after by researchers in an adjoining room.

### Infant video activation paradigm

Mothers were exposed to an 8-min fixed sequence design and scanned while viewing 30-sec blocks of continuous video of: (i) their own infant; (ii) emotionally neutral stimuli (moving traffic); (iii) an unfamiliar infant (which was the infant of another participant in part of a larger study); (iv) repeat neutral block. This 2-min sequence was repeated 4 times, though to minimise habituation effects, each 30-sec block contained 2 edited clips which were alternated in order with each repetition. The ‘own infant’ and ‘unknown infant’ blocks showed a head-and-shoulders view of the infant (who was in a sitting position, with a black background) with neutral to mildly positive affect. That is, we avoided including any negativity and all extremes of affect and motion, while allowing for a sufficient range so that the video ‘felt’ naturalistic, since extended neutral affect tended to be accompanied by extended stillness and/or conveyed boredom which would be unlikely to be perceived as affectively neutral by mothers. Frames in which the face is occluded by limbs, clothes etc. were also excluded. Own and unknown infant were matched for infant gender (100%) and well matched for ethnicity (Caucasian or non-Caucasian; 90%), appearance of the infant's age, and degree of expressivity and movement. As in our previous study [12], the neutral stimuli comprised of moving traffic from a stationary viewpoint representing moving imagery with no emotional content. Mothers were asked to view these videos just as if they were watching television – the intention of this instruction was to normalise the experience for mothers and reduce their anxiety regarding what they ought to be doing. There was no sound, and no responses were required.

### fMRI imaging protocol

Participants underwent fMRI imaging using a 1.5 T Philips Intera MRI scanner (Philips Healthcare, The Netherlands). Ninety-six volumes were acquired with T2*-weighted gradient echo-planar imaging. Repetition time was 2.55 s, and echo time was 40 ms. Each volume comprised 34 ascending axial sections at a section thickness of 4.5 mm with a 0.5 mm gap and in-plane resolution of 3.5×3.5 mm. A T1-weighted structural image was acquired for use in spatial preprocessing and was examined to exclude participants with any structural abnormalities (no abnormalities were observed).

### Behaviour and reported measures

#### The Mothers' Object Relations Scale-Short Form (MORS-SF) [23]

This questionnaire was developed as a screening tool for identifying potential areas of difficulty in the early mother-infant relationship derived from studying mothers' narrative accounts of their perceptions of infant's feelings, cognitions, and behaviours. The MORS-SF items have been shown to possess stable and internally coherent scales, and measures 2 factors: ‘warmth’ and ‘invasiveness’. In the current study, we were interested in the warmth factor in an attempt to capture the mother's emotional ‘bond’ or positivity toward her infant, by asking her about her perceived infant's warmth toward her, as this is thought to be less subject to social desirability effects than asking about her feelings about her infant. This factor comprises 7 items to which mothers respond on a 0 (never) to 5 (always) scale.

#### Manchester Assessment of Caregiver-Infant Interaction (MACI) [24], [25]

Six-minute videos of caregiver-infant interaction were evaluated by a trained, reliable rater on 7 MACI scales which globally assess core characteristics of caregiver-infant play interaction on a 7-point scale. Each clips was reviewed several times to arrive at a rating for each scale, taking into account all sources of information in relation to quantity and quality of behaviours. The MACI has been used in healthy mothers with typically developing infants and in infants with an older sibling with autism [24], [25], with good to excellent psychometric properties, and inter-rater reliability. The measure also shows moderate stability in caregiver behaviour and mother-infant mutuality between 7 and 14 months in healthy mother-infant dyads [25], which provides face validity that this measures an ‘interaction pattern’, though some change is expected over this relatively long period of infancy. Ratings have been reported to be independent of infant gender, infant non-verbal development, and maternal age [24], and pilot findings suggest stability across home and lab contexts [26].

Using independent blind ratings of 50% clips in the sample, high inter-rater agreement was shown in all reported scales (single-measures intraclass correlations using a two-way mixed effects model; absolute agreement definition): caregiver sensitive responsiveness: *r* = 0.79 (p = 0.003); caregiver nondirectiveness: *r* = 0.74 (p = 0.005); infant attentiveness to parent: *r* = 0.83 (p = 0.001); infant positive affect: *r* = 0.76 (p<0.001).

### Statistical analysis

Functional imaging data were analysed using statistical parametric mapping (SPM5; Wellcome Dept of Cognitive Neurology, London, UK). Images were realigned to correct for motion using the first image as a reference image. Co-registration of the structural (T1-weighted) and functional (T2*-weighted) images was performed. Images were spatially normalised into a standard stereotactic space using non-linear transformation and were smoothed using a Gaussian kernel filter of full width at half maximum of 3 times the voxel size (i.e. 10.5, 10.5 and 13.5 mm). First level analysis (fixed effects) was performed on each participant to generate a mean blood oxygenation level dependent (BOLD) image for each of the following contrasts: (1) own infant versus other infant; (2) other infant versus own infant; (3) all infants versus neutral stimuli.

At second level (random effects) analysis, we assessed whole brain BOLD response changes observed at a false discovery rate p<0.05 for the (own and unknown) infant video stimuli compared to neutral control stimuli, and at an uncorrected p value of <0.001 for the contrasts of viewing own infant compared to viewing an unknown infant, and unknown infant compared with own infant. Given that the amygdala response is one of the most consistent findings in previous, similar studies [4], [7], [10], [12], we used region of interest (ROI) analysis in this region. A small volume correction (10 mm sphere, p<0.05 Family Wise Error (FWE) corrected) was performed based on the coordinates for the amygdala response from the same fMRI paradigm previously published [12], which this study had set out to replicate. To check whether these BOLD changes were due to activation in response to viewing own versus unknown infant, and not due to de-activation (that was lesser compared to the unknown infant condition), a time course analysis was conducted of the mean BOLD response in a region which showed the largest effect. This was extracted using an ROI analysis of individual participants' BOLD responses under each condition in an 8 mm ROI centred on these coordinates.

A second exploratory whole brain analysis and small volume correction of the amygdala were conducted adjusting for current breastfeeding status as a binary variable. This was to take into account the possibility that breastfeeding, which is linked with oxytocin level [27], may affect maternal neural response to their infant [28], and in this study, 9 mothers reported that they currently breastfed. However, this exploratory analysis was performed recognising particular substantial caveats: (1) Our correlational analyses (see below) were concerned with relating mothers' brain responses to viewing their own infants with behavioural and reported correlates (e.g. maternal responsiveness); therefore we may not wish to adjust statistically for breastfeeding (or inferred oxytocin level), as oxytocin may explain any brain-behaviour association that might disappear if breastfeeding is controlled for; (2) Our information on breastfeeding may be inadequate to judge oxytocin level as we lack information on the interval between breastfeeding and imaging (oxytocin level varies significantly as a function of the breastfeeding cycle [27]) and whether breastfeeding mothers did so exclusively; and (3) These analyses are likely to be underpowered as the study was not designed to examine the effects of breastfeeding; therefore, these analyses are presented as supplementary in support of the main analysis.

Correlational analyses consisted of relating the main effects found in the own infant versus unknown infant contrast (unadjusted analyses) with key mother-infant relations measures, adjusting for infant age and the mother's educational level (a proxy of socioeconomic status). From the MACI, most of the scales were inter-correlated (see Appendix S1) as we might expect given the inter-related nature of social interactions and our previous findings in healthy samples [25]. Of the MACI, the current study utilised two scales of observed caregiver behaviour and a composite scale of observed infant behaviour. The caregiver scales were: (i) ‘Sensitive responsiveness’ (caregiver behaviours that appropriately and promptly address the infant's identified behaviours (and lack of behaviour) at the service of meeting the infant's moment-to-moment and developmental needs, in contrast to low-sensitive responses and/or a lack of responding); (ii) ‘Nondirectiveness’ (a behavioural and mental ‘acceptance’ or centeredness on the infant's experience in contrast to implicitly and explicitly demanding, intrusive and negative behaviours that may restrict the infant's activity and autonomy). Although the two scales are conceptually related especially at the high end, we examined them separately since low sensitively responsive caregivers could have a low or high nondirectiveness rating, corresponding to a more controlling or more passive interactive style respectively, which may be differentiated at a neural level. A third MACI scale was a composite mean rating of infant attentiveness (the amount and quality of interest in, and visual contact with, the caregiver as opposed to focus on other stimuli or self-absorption) and infant positive affect (the extent to which the infant shows positive affect in comparison to negative affect), which were inter-correlated (r = .56; p = 0.01). This infant composite was studied on the basis that maternal neurocorrelates with maternal behaviour may also be associated (indirectly) with infant behaviour if maternal interactive behaviours, which relate to brain responses to viewing own infant, impact on the infant's interactive behaviour. MORS-SF Warmth, which was not associated with any behavioural interaction scale in the current sample, was a mother-reported measure of the infant's warmth toward the mother.

## Results

### Main Effects

To examine the neural brain response to viewing infants compared to emotionally neutral moving stimuli, the infant videos (own infant and unknown infant) were compared to neutral (traffic) video. Significant BOLD responses were found in bilateral visual processing regions (BA 18, 19) including the fusiform face area (BA 37), as well as middle and superior temporal gyri (BA21, 38, 39), cerebellum, parietal regions (inferior parietal lobule, precuneus, postcentral gyrus) and middle and superior frontal gyri (p<0.05 FDR) (*Table 2*). Significant responses following whole brain family wise error correction (p<0.05) were detected in bilateral fusiform gyrus, cerebellum, amygdala, middle temporal gyrus and occipital regions (inferior occipital gyrus, cuneus).

**Table 2:** Areas of significant activation from all infants minus neutral contrast (FDR corrected p<0.05).

Region activated	Left/Right	Brodmann's Area	Cluster size (k)	Talairach coordinates			P value
				X	Y	Z	
Cerebellum/Inferior Occipital Gyrus	L	19	867	−41	−50	−18	0.001*
Cerebellum	R		138	0	−55	−24	0.006
				2	−63	−20	0.008
				9	−77	−20	0.019
			15	34	−77	−26	0.025
				25	−84	−26	0.043
Cuneus	L	18	145	−9	−96	8	0.004*
Middle Temporal Gyrus	L	21	14	−44	10	−31	0.012
	R	39	600	54	−60	7	0.006*
Fusiform Gyrus	R	37		48	−54	−17	0.008*
				39	−46	−18	0.04*
Inferior Parietal Lobule	R	40	95	61	−32	24	0.001
Postcentral Gyrus	R		2	59	−29	42	0.004
Precuneus		31	102	0	−52	32	0.002
Middle Frontal Gyrus	R	46	14	41	32	13	0.002
		9	143	48	11	36	0.003
		6		41	9	51	0.024
Superior Frontal Gyrus	R	8	57	7	18	54	0.01
	R	6		7	30	54	0.014
	Mid	9	117	0	55	27	0.004
Medial Frontal Gyrus	Mid	10		0	56	19	0.004
Superior Temporal Gyrus	R	38	90	25	8	−24	0.005
				34	16	−28	0.026
Amygdala*	R			18	−7	−13	0.017
Middle Occipital Gyrus	R	18	15	11	−93	16	0.008
Anterior Cingulate	L	32	10	−16	30	13	0.011
Cingulate Gyrus	R	23	13	2	−12	23	0.017
Cerebellum	L		10	−16	−66	−14	0.019
Inferior Frontal Gyrus	R	47	14	28	28	−18	0.021
	L	47	33	−39	20	−14	0.02
Insula	L	13	18	−39	18	3	0.02
	L			−41	11	−4	0.028
	R			−39	10	18	0.022
Uncus	L	28	14	−23	8	−21	0.021

* FWE p<0.05.

**Figure 1:**
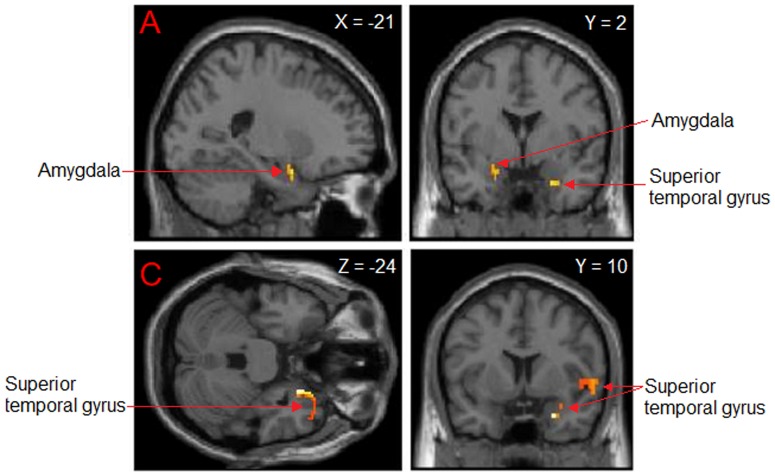
Regions of maternal brain activation in response to viewing own infant versus unknown infant: (A) Amygdala (SVC FWE; p<0.05) in sagittal view, and coronal section with right superior temporal activation also visible; (B) superior temporal gyrus in horizontal and coronal view (p<0.001 uncorrected) viewed from the right (see also *Figure 2*).

**Figure 2:**
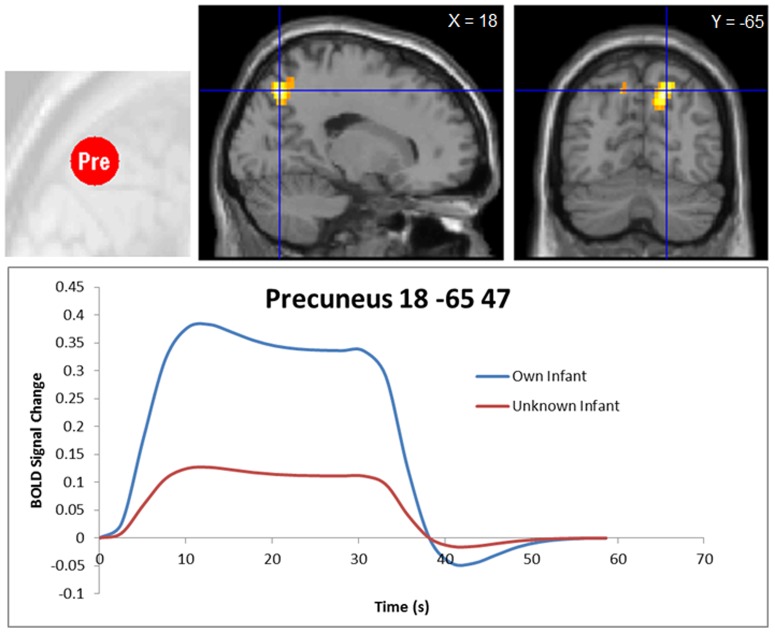
Time course of the group mean BOLD response in the precuneus (18, −65, 47) for viewing video of own infant and unknown infant for 30 secs.

No significant differences emerged between viewing own infant minus unknown infant, or vice versa, after false discovery rate whole brain correction. To assess whether there were more subtle differences between these very similar stimuli (matched closely in infant visual characteristics), the threshold was lowered to a *p*<0.001 uncorrected. We report these findings with caution, noting that interpreting uncorrected results is compromised and must be considered in context. Mothers viewing own infant compared to an unknown infant had enhanced BOLD responses in the bilateral precuneus, right superior temporal gyrus, right medial (BA 8 and 9) and left middle (BA 6) frontal gyri (p<0.001 uncorrected) (*Table 3* *;* *Figures 1* *and* *2*). There was also cerebellar, and superior and inferior parietal activation. Based on the co-ordinates from our previous findings [12], ROI analysis showed Family-Wise Error (FWE) corrected (p<0.05) activation in the left amygdala for mothers viewing their own infant>unknown infant. A time course analysis of the mean BOLD response in the precuneus showed a relative increase in BOLD signal in the group mean for the duration in which the infant video was presented compared to neutral stimuli, and an enhanced response to own infant (*Figure 2*).

**Table 3:** Areas of significant activation in own>unknown infant contrast.*

Region activated	Left/right	BA	Cluster size (k)	Talairach coordinates (x y z)			Z score
Own infant minus unknown infant							
Cerebellum	R		27	25	−61	−17	4.36
	L		52	−41	−56	−21	3.89
	L		12	−44	−66	−20	3.75
	L		13	−16	−44	−18	3.79
	Mid			0	−54	−17	3.56
Precuneus	R	7	32	18	−65	47	4.21
	R			11	−71	48	3.19
	L		24	−12	−51	50	3.89
Superior Temporal Gyrus	R	38	15	25	10	−24	4.18
Middle Frontal Gyrus	:	6	26	−39	−2	48	4.04
Medial Frontal Gyrus	R	9	12	21	44	16	4.15
	R	8	67	2	26	39	3.9
Superior Parietal Lobule	L	7		−16	−52	58	3.67
Inferior Parietal Lobule	R	40	12	39	−40	50	3.74
Middle Occipital Gyrus	L	18	11	−28	−78	1	3.72
Postcentral Gyrus	R	1	13	59	−25	42	3.6
Precentral Gyrus	R	6	26	54	−5	33	3.52
	L	4	11	−41	−12	41	3.5
Amygdala**	L		5	−21	2	−20	3.03
Unknown infant minus own infant							
Middle Temporal Gyrus		21	59	66	−24	−2	4.2
				66	−33	−2	3.72
				59	−25	−9	3.39
Cerebellum			16	46	−67	−34	3.99
Medial Frontal Gyrus		10	35	11	35	−12	3.9
		11	13	−5	39	−12	3.57
Subcallosal Gyrus		11	13	−12	24	−11	3.78

* *p*<0.001 uncorrected.

** SVC FWE p<0.05 5 mm radius from previous coordinates (−21, 0, −18) [12].

In response to the unknown minus own infant contrast, there was enhanced right middle temporal (BA21) and bilateral medial frontal (BA 10 and 11) activation (*p*<0.001 uncorrected) (*Table 3*).

To explore the possibility that breastfeeding confounded our findings (see statistical analysis), the own infant versus unknown infant contrast was re-analysed adjusting for current breastfeeding status. Neural response to this contrast after controlling for breastfeeding was retained in the precuneus, cerebellum, middle frontal gyrus, and superior frontal gyrus (*Table 4*). However, left inferior frontal and right middle temporal activation was also found, and there was no significant amygdala activation.

**Table 4:** Areas of significant activation in own>unknown infant contrast* controlling for breastfeeding.*

Region activated	Left/right	BA	Cluster (K)	X	Y	Z	Z score
Precuneus	R	7	73	18	−70	44	3.87
	R	19		32	−70	40	3.2
Cerebellum	L		302	−23	−37	−22	3.78
Temporal sub-Gyrus	L			−32	−30	−12	3.38
Cerebellum	L			−32	−41	−25	3.28
Middle Frontal Gyrus	L	46	93	−41	25	21	3.54
Inferior Frontal Gyrus	L	45		−50	23	14	3.21
Middle Temporal Gyrus	L	39	87	−35	−63	25	3.51
Inferior Frontal Gyrus	L	47	47	−35	15	−18	3.49
	L	47		−23	15	−18	3.2
Superior Temporal Gyrus	R	22	17	48	−2	0	3.37
Postcentral Gyrus	R	2	48	50	−22	45	3.32
	R	3	23	63	−19	34	3.2
Middle Temporal Gyrus	R	21	15	59	4	−14	3.12

*p<0.001 uncorrected.

### Behavioural and reported correlates

Our relational measures – both observed (MACI maternal sensitive responsiveness, maternal nondirectiveness and infant interactive behaviour) and reported (MORS-SF Warmth) – did not vary according to key sociodemographic and maternal characteristics (mother's ethnicity, professional occupation, infant gender, or current breastfeeding status), except that mothers with sons were more directive than mothers with daughters in our sample (*F* = 4.67; *p* = 0.05).

Correlations between the relational measures and brain responses should be viewed cautiously considering the subtle differences between stimuli and the low comparability of behavioural correlates. With respect to mother-infant interaction, caregiver nondirectiveness, but not caregiver sensitive responsiveness, was associated with several regions activated when viewing own infant minus unknown infant: lingual gyrus, left putamen, and right inferior and middle frontal gyri (BA9 and BA46; p<0.001 uncorrected) (*Table 5*). Infant interaction was associated with BOLD increases in the middle frontal gyrus, bilaterally, and with the right precentral gyrus and left thalamus (p<0.001 uncorrected). Mothers' perceived warmth of her infant was correlated with several regions activated when viewing own minus unknown infant in the right and midline precuneus, bilateral cuneus, right middle occipital gyrus, medial frontal gyrus, insula, and cerebellar regions (p<0.001 uncorrected).

**Table 5:** Behavioural and mother-reported correlates with own>unknown infant contrast.*

Correlate	Region activated	Cluster (k)	BA	x	y	z	Z Score
Maternal nondirectiveness	Lingual/fusiform gyrus	41	18	−16	−81	−9	3.45
	Putamen	17		−16	5	7	3.43
	Inferior Frontal Gyrus	48	9	41	3	29	3.43
	Middle Frontal Gyrus	28	46	41	30	21	3.16
Infant interactive behaviour	Middle Frontal Gyrus	68	9	44	9	36	3.65
		32	6	−30	7	51	3.25
	Precentral Gyrus	22	4	46	−9	41	3.21
	Thalamus	20		−16	−13	4	3.10
Mother reported warmth	Precuneus	113	7	7	−65	36	4.13
			7	0	−65	44	3.59
	Cuneus		7	0	−67	33	3.82
	Cuneus	55	17	−12	−91	8	3.94
	Cuneus	105	19	18	−90	23	3.94
	Middle Occipital Gyrus		19	28	−87	15	3.23
	Pyramis	53		9	−82	−23	3.26
	Medial Frontal Gyrus	24	10	−2	56	12	3.26
	Claustrum	15		21	27	6	3.24
	Insula	18	13	34	−28	20	3.21

*p<0.001 uncorrected.

## Discussion

In one of the largest samples of healthy new mothers to date, the current study explored the brain regions that respond when mothers viewed videos of their own infant relative to an unknown infant, and whether such unique neural responses were associated with behavioural and self-reported measures of mother-infant relations. Firstly, acknowledging the large discrepancies in findings across previous comparable studies, we conducted exploratory whole brain analyses (except for an ROI in the amygdala) and found subtle but significant differences in activation patterns when mothers viewed videos of their own compared to an unfamiliar well-matched infant. These included the parietal areas (bilateral precuenus, left superior parietal lobule, right inferior parietal lobule), right superior temporal gyrus, left middle frontal gyrus, right medial frontal gyrus, left middle occipital gyrus, left amygdala, and primary motor and somatosensory areas (cerebellum, precentral gyrus, postcentral gyrus). Our findings represent a partial replication of earlier studies (including our own [12]), which also reported enhanced left amygdala, middle occipital and cerebellar activity in the own versus unknown infant contrast. Secondly, we found that greater own-infant response (relative to unknown infant) in the middle frontal gyrus was associated with higher quality play interactions, while greater sensory and visual area activations, and to a lesser extent, insula activations, were associated with greater perceived maternal warmth towards her infant.

### Viewing own minus unknown infant

When mothers viewed their own infant, the differential engagement of the parietal, frontal, primary motor and somatosensory regions implicates recruitment of areas involved in the processing and integration of visual information, and the frontoparietal mirror neuron system that is involved in empathy [29]. Left amygdala activation was also enhanced, consistent with several previous studies [4], [7], [10], [12], including those involving infant cry stimuli [30], [31], and is associated with anxiety, fear and preoccupation [32]. Our findings triangulate with longitudinal evidence suggesting grey matter volume increases in the prefrontal, parietal and midbrain regions through the early postpartum [33]. In line with previous studies [7], [10], [11], we also found a precuneus response, which – along with the left middle frontal gyrus – was recently shown to be associated with empathic accuracy in inferring others' mental states from videos in healthy adults but not in schizophrenia patients [34]. In other studies, the precuneus is activated when adults evaluate their own or other's emotional states [35] and make empathic judgements [36], and when nulliparous women view sad (compared with neutral) infant faces [37].

However, the differences in neural processing of own infant versus unknown infant were more subtle than most previous studies. This was perhaps expected, given the more subtle visual differences between infant stimuli conditions than used in most studies. Our aim was to distil the biological underpinnings of the mother “being with their infant” rather than viewing their infant in particular conditions (e.g. play with mother, separation, smiling), which tends also to introduce a high degree of variability in the stimuli. Thus, our findings may be taken to reflect a relatively subtle pattern of neural response as part of the mother's ‘default’ emotional bond with her infant, rather than in response to more emotionally evocative stimuli.

In further exploratory analyses, our main findings for the own infant versus unknown infant contrast were robust even when current breastfeeding was controlled for with the exception of the amygdala which was no longer significant. As the amygdala ROI was based on previous findings not accounting for breastfeeding, this is not surprising with a discreet amygdala response. Given the relative lack of power for controlling for breastfeeding and the lack of specificity/detail in the binary breastfeeding variable, caution must be taken in drawing firm conclusions from this supplementary analysis. However, taking into account such limitations, additional BOLD signal changes were found in the left inferior frontal gyrus and the middle temporal gyrus. These findings may provide directions for future research in that the right middle temporal gyrus is understood to be involved in the processing and interpretation of familiar faces [38], [39] and was a region of activationin other similar fMRI studies [9], [11]. The left inferior frontal gyrus (along with the precuneus) is implicated in the regeneration of rich episodic contextual associations [40] and activation has been found in previous studies, though on the right rather than left side [7], [9], [11]. Restraint must be taken in comparing these analyses given that the study was not sufficiently powered to address the impact of breastfeeding explicitly, which would require a larger study and more detailed breastfeeding information.

No differential response to viewing own infant was found in some of the brain regions most often reported previously to be activated in this contrast, namely the orbitofrontal cortex [11], [12], superior frontal gyrus [5], [7], [10] and right amygdala [4], [10]. However, in the current study, these areas were recruited in response to infants generally (own and unknown) compared to neutral stimuli. The patterns of brain activation in our sample did not differentiate between infants as much as other studies; perhaps because we did not use as emotive stimuli as others have done (which may be important for ‘activating’ the mother's ‘emotional attachment’ system). In addition, the own infant and control stimuli used in previous studies may have differed substantially in behaviour and emotiveness (whereas videos in the current study were limited in behavioural repertoire and we matched on general activity level). Previous studies have also reported activation in the left thalamic areas in response to own infant, but only when viewing them in highly emotive contexts [5], [9], [11]. Here, we also found a moderate correlation between a left thalamic response and infant interactive behaviour. This suggests that mothers with more thalamic activation in response to viewing their infant may have more positive, social infants generally (although expressivity was controlled for to a large degree in the fMRI stimuli).

### Correlates with mother-infant relational variables

Our secondary objective explored links between the mother's unique neural responses to her own infant and concurrent mother-infant relational behaviour and maternal perceptions of relational warmth. Moderate correlations were reported at an uncorrected threshold of p<0.001; these findings should be considered preliminary and may partially reflect, for the behavioural measures, their relatively limited (1 to 7) scale (although our sample showed a wide distribution). Greater own-infant response in the middle frontal gyrus – which is implicated in empathy [29], [34] – was associated with higher quality play interactions (less directive parent behaviour and more infant interactive behaviour). Less directive interactions were also associated with greater lingual gyrus and putamen activation, which are implicated in visual and motor processes, respectively. However, correlations with mothers' perceived infant warmth were focused around the sensory and visual areas, and to a lesser extent, the insula. Taken together, these patterns may suggest that enhanced frontal activation unique to viewing own infant may not be associated with mother's reported relational warmth (which is emotionally based, as perceived by the mother), but may be related to less directive caregiver behaviour (which is likely to require social cognitive, planning and other executive functioning skills; e.g. facilitating autonomy, inhibiting directive/negative responses, actively following the infant's experience) and more positive infant interaction as a result.

Finally, our behavioural measure of ‘maternal sensitive responsiveness’ did not correlate with neural activation patterns to own infant. It is possible that in the current sample, nondirectiveness (that is, an ‘accepting’ focus on the infant experience as opposed to controlling, intrusive and demanding behaviour) was a more sensitive indicator of overall maternal sensitivity than level of sensitive responsiveness. All mothers who were rated low in sensitive responsiveness were low in nondirectiveness suggesting that all low sensitively responsive mothers in this sample tended to be directive/controlling (rather than passively non-responsive).

### Strengths and Limitations

Strengths of this study were the meticulous use of video stimuli and matching with the unknown infant, and the comparatively large sample size. Although this is one of the largest fMRI studies of new mothers to date, the sample only allowed for a correlational analysis to examine links between fMRI and mother-infant relational data. A larger sample would allow for mothers with optimal interactions to be contrasted with those with less optimal interactions, with potential for distinguishing withdrawn from intrusive mothers who may differ in their neurobiological profiles. Although we attempted to control for current breastfeeding, this was limited due to lack of statistical power and lack of information regarding the time at which mothers last breastfed, but may be linked to oxytocin levels that may have affected maternal neural response to their infant. There was no ‘other familiar infant’ condition in our fMRI paradigm; this would allow for clearer distinction of infant recognition effects from relational/affiliative effects in the own infant versus unknown infant contrast. Our fMRI paradigm did not include a resting period as a contrast, as we chose to use a neutral non-infant stimulus as our baseline, controlling for visual stimulation. The use of more discrete, microanalytic measures of maternal behaviour (e.g. positive affect, social gaze, frequency of identification of infant behaviour irrespective of sensitivity of response) during more naturalistic daily tasks (e.g. feeding) may prove to be more closely related to neural responses unique to ones own infant. In future, procedures that activate the mother's attachment system (more emotive fMRI stimuli), as well as the infant's attachment system (a more challenging context in which to capture mother-infant interaction), may elicit greater differentiation between conditions/participants that may better link the parental brain with behaviour. Finally, the inclusion of post-scan interviews may have provided additional helpful information on the mothers' experience of viewing the video clips in the scanner.

### Conclusion and implications

This study partially replicates previous studies and the findings suggest that new mothers show subtle patterns of neural response specific to viewing their own infant, which primarily involve the parietal-frontal-visuospatial and amygdala regions. These findings largely remained even after controlling for current breastfeeding status, with the exception of the amgydala. The magnitude of frontal activation appears to be associated particularly with the quality of mother-infant interactive behaviour, while the increased recruitment of the sensorimotor areas and insula was associated with maternal perceived warmth.

Our findings suggest that it would be valuable to test more specific hypotheses to explore the neural substrates involved in women at risk of suboptimal parenting, or who have difficulties responding to, and bonding with, their infant. For example, the current findings might be taken to imply that directive (intrusive or controlling) mothers show reduced recruitment of the frontal areas associated with cognitive empathy, rather than the enhanced response of anxiety-related regions.

Further work is needed to explore the possible affective cognitive pathways to low maternal sensitivity using more sophisticated infant fMRI stimuli and measures of behaviour and affective cognition. This may be particularly relevant in some mothers with specific clinical deficits in affective cognition, such as mothers with depression and other mental disorders who may have difficulties bonding [41], [42] and are more likely to show disrupted maternal sensitivity [43], [44]. This fMRI paradigm could be developed further as a biomarker of longitudinal change [45] in order to complement behavioural outcomes in response to parenting intervention [46].

## Supporting Information

Appendix S1 **Correlations between MACI scales (N = 20).**
(DOCX)
