# Supplementary material for: The Neural Basis of Maternal Bonding
Source: PLoS One. 2014 Mar 4;9(3):e88436. doi: 10.1371/journal.pone.0088436 (PMC3942310; doi:10.1371/journal.pone.0088436)
Supplement: Appendix S1 — Correlations between MACI scales (N = 20). (DOCX) [file pone.0088436.s001.docx]

**Appendix**

**Table 1. Correlations between MACI scales (N = 20)**

|  | Caregiver  sensitive responsive | Caregiver nondirective-ness | Infant attentive-ness | Infant positive affect | Infant liveliness | Dyadic mutual-ity |
| --- | --- | --- | --- | --- | --- | --- |
| Nondirectiveness | .90** |  |  |  |  | . |
| Attentiveness | .87** | .80** |  |  |  | . |
| Positive affect | .56** | .55** | .59** |  |  | . |
| Liveliness | .47* | .54** | .28 | .11 |  |  |
| Mutuality | .90** | .84** | .86** | .60** | .55** |  |
| Intensity of engagement | .85** | .78** | .79** | .57** | .57** | .90**. |

*p < 0.05; **p<0.01
